# CXADR polymorphism rs6517774 modifies islet autoimmunity characteristics and exhibits sex disparity

**DOI:** 10.3389/fgene.2023.1248701

**Published:** 2023-11-02

**Authors:** Lucas Nygård, Milla Valta, Antti-Pekka Laine, Jorma Toppari, Mikael Knip, Riitta Veijola, Heikki Hyöty, Jorma Ilonen, Johanna Lempainen

**Affiliations:** ^1^ Immunogenetics Laboratory, Institute of Biomedicine, University of Turku, Turku, Finland; ^2^ Department of Clinical Microbiology, Institute of Clinical Medicine, University of Eastern Finland, Kuopio, Finland; ^3^ Research Centre for Integrative Physiology and Pharmacology, and Centre for Population Health Research, Institute of Biomedicine, University of Turku, Turku, Finland; ^4^ Department of Pediatrics, Turku University Hospital, Turku, Finland; ^5^ Research Program for Clinical and Molecular Metabolism, Faculty of Medicine, University of Helsinki, Helsinki, Finland; ^6^ Pediatric Research Center, New Children’s Hospital, Helsinki University Hospital, Helsinki, Finland; ^7^ Tampere Center for Child Health Research, Tampere University Hospital, Tampere, Finland; ^8^ Medical Research Center, Department of Pediatrics, PEDEGO Research Unit, University of Oulu, Oulu, Finland; ^9^ Department of Children and Adolescents, Oulu University Hospital, Oulu, Finland; ^10^ Faculty of Medicine and Health Technology, Tampere University, Tampere, Finland; ^11^ Fimlab Laboratories Ltd., Pirkanmaa Hospital District, Tampere, Finland; ^12^ Clinical Microbiology, Turku University Hospital, Turku, Finland

**Keywords:** type 1 diabetes, SNP, CXADR, islet autoimmunity, sex disparity, autoantibodies, HLA

## Abstract

Enteroviral infections have been linked to the development of islet autoimmunity (IA) and type 1 diabetes (T1D), and the coxsackie and adenovirus receptor (CXADR) is one of the ligands used by adenoviruses and enteroviruses for cell internalization. Two *CXADR* single nucleotide polymorphisms (SNPs), rs6517774 and rs2824404, were previously associated with an increased susceptibility to IA in the international TEDDY study (The Environmental Determinants of Diabetes in the Young). This study aimed to replicate the results by genotyping 2886 children enrolled in the Finnish Diabetes Prediction and Prevention study (DIPP). In our preliminary analysis of the SNPs’ allelic distributions, we could not find any association with IA susceptibility. However, a stratified analysis revealed a sex disparity, since the allelic distribution of rs6517774 was different when comparing autoantibody positive females with males; a difference not seen in healthy subjects. By using HLA risk groups and sex as covariates, a Cox regression survival analysis found that the rs6517774 (A/G) SNP was associated with a lower age at seroconversion in females (Female*rs6517774-AA; HR = 1.53, *p* = 0.002), while introducing a protective effect in males. Accordingly, we propose that rs6517774 alters IA characteristics by modifying the age at seroconversion in a sex-dependent manner. In light of this observation, rs6517774 now joins a limited set on SNPs found to introduce sex-dependent risk effects on the age at IA initiation.

## 1 Introduction

Type 1 diabetes (T1D) has a multifactorial etiology, wherein both genetic and environmental factors predispose children to disease. HLA class II alleles, together with polymorphisms in other HLA genes and variants in more than 60 non-HLA loci, constitute the principial risk factors for disease development (Ilonen et al., 2019). Among environmental factors, both viral infections and dietary factors have been implicated to be associated with IA and T1D (Ilonen et al., 2019).

There is a strong association between T1D and enterovirus infections (Wang et al., 2021; Yang et al., 2021), and specific human adenoviruses and enteroviruses, especially species B enteroviruses (EV-B), have an inherent tropism for β-cells (Roivainen et al., 2000; Ylipaasto et al., 2004). The coxsackie and adenoviral receptor (CXADR) enables the internalization of human adenoviruses and enteroviruses (Karttunen et al., 2003; Ifie et al., 2018; Herrmann et al., 2020). Thereto, the CXADR functions as a tight-junction protein that facilitates cellular adhesion (Park et al., 2021), and the receptor is essential for normal embryonal development (Excoffon, 2020; Sharma et al., 2021). Moreover, the CXADR is expressed by multiple cell types throughout the body, including respiratory epithelial cells, myocardial cells and pancreatic β-cells (Zanone et al., 2007; Excoffon, 2020; Owczarek et al., 2022). Lastly, in the context of disease pathology, enhanced expression of CXADRs has been observed in the pancreatic cells of individuals with T1D (Hodik et al., 2016), and a range of *CXADR* SNPs are associated with myocardial infarction (Marsman et al., 2011) and lung cancer (Li et al., 2022).

Recently, the multinational TEDDY-study (The Environmental Determinants of Diabetes in the Young) reported that the *CXADR* rs6517774-G SNP correlated with a lower occurrence of EV-B positive stools in children with a genetic predisposition for T1D (Vehik et al., 2019). Moreover, through an additive effect, the minor rs6517774-G allele was associated with a lower occurrence of EV-B infections concurrent with an increase in the odds of IA development. The *CXADR* rs2824404-C SNP was also associated with IA, but only in Finnish subjects (Vehik et al., 2019). Furthermore, the study reported that Finnish children carrying both minor alleles of rs6517774-G and rs2824404-C were more likely to develop IA also in the absence of EV-B (Vehik et al., 2019). The authors concluded that these findings should be studied independently in the Finnish population. Therefore, the current study intended to validate the previous findings in an independent cohort and aimed to further determine how the *CXADR* polymorphisms modify a broader range of IA characteristics in Finnish children enrolled in the Diabetes Prediction and Prevention study (DIPP).

## 2 Materials and methods

### 2.1 Study participants and cohort characteristics

The DIPP study (ClinicalTrials.gov ID: NCT03269084) is a longitudinal prospective study initiated in 1994 that has been screening newborn infants for genetic predisposition to T1D at the university hospitals in Turku, Tampere, and Oulu (Mikk et al., 2020; ClinicalTrials.gov, 2023). Thus, parents of children born with a T1D associated HLA genotype can opt to enroll their offspring into a follow-up program. Starting from the age of 3 months, the follow-up includes regular screenings for islet-specific autoantibodies (AAb) (Parikka et al., 2012). The study has been approved by the ethics committee of the Northern Ostrobothnia Hospital District and is conducted in accordance with the Declaration of Helsinki. Informed consent was acquired by the subjects’ legal guardians prior to screening and recruitment to follow-up study.

The cohort analyzed here included 2886 Finnish children, born between 1994 and 2015, with serological data and DNA samples in the DIPP study repository (Table 1). The seropositive cases (*N* = 976) were all positive for at least one persistent (defined as two consecutive samples taken at an interval of 2–3-months) islet-specific AAbs during the follow-up. The studied AAbs were insulin autoantibodies (IAA), insulinoma-associated protein 2 autoantibodies (IA-2A), glutamic acid decarboxylase 65 autoantibodies (GADA) and zinc transporter 8 autoantibodies (ZnT8A) (Juusola et al., 2016; Pöllänen et al., 2019). The controls (*N* = 1910) were children enrolled in the DIPP study that have not presented AAbs or developed T1D during the follow-up period. The cohort included HLA-genotyped risk groups ranging from class 0 to 5, with high-risk (class 5; OR = 13.23), moderately increased risk (class 4; OR = 7.20) and slightly increased risk (class 3; OR = 1.94) groups accounting for 95% of the seropositive cases (Ilonen et al., 2016). The protective, slightly protective, and neutral risk groups (annotated 210) were pooled into one group due to their rarity (4%) in seropositive cases. The case-control pairs were matched according to the cases’ sex, study center and date of birth (± 2 months). Moreover, in the survival analysis with subgroups, case-control pairs were matched according to following AAb characteristics: IAA as the first AAb, GADA as the first AAb, or all groups combined. The distribution of HLA and islet autoimmunity characteristics in this study cohort has been summarized in Table 1. Lastly, to the readers’ discretion, a subset of cases and controls were excluded from the final subgroup analyses due to incomplete genotype or AAb data.

**TABLE 1:** A. The distribution of islet autoimmunity characteristics in AAb-positive cases, and HLA group distribution in AAb-positive cases and seronegative controls. B. Age at seroconversion stratified for IAA-only first positives and GADA-only first positives. The distribution of sex (% of males) is presented when applicable.

A	Cases (%)	Males (%)	Controls (%)	Males (%)
No. of subjects per group	976	59.5	1910	59.7
Islet autoimmunity characteristic				
One AAb during follow-up	416 (42.6)	59.9		
Two AAbs during follow-up	246 (25.2)	58.5		
Three or more AAbs during follow-up	314 (32.2)	59.9		
Sum	976	59.5		
IAA as the first detected AAb	330 (33.8)	57.0		
GADA as the first detected AAb	329 (33.7)	60.8		
IA-2A as the first detected AAb	52 (5.3)	69.2		
ZnT8A as the first detected AAb	17 (1.7)	70.6		
Other single AAb	12 (1.2)	66.7		
Multiple AAbs at first detection	236 (24.2)	58.1		
Sum	976	59.5		
Age at seroconversion, years				
≤2	366 (37.5)	61.2		
>2 and ≤4	254 (26.0)	58.3		
>4 and ≤6	148 (15.2)	53.4		
>6 and ≤10	147 (15.1)	62.6		
>10 and ≤15	61 (6.2)	62.3		
Sum	976	59.5		
No. of subjects per HLA risk group				
HLA risk groups 0, 1, and 2	39 (4.0)	51.3	194 (10.2)	62.4
HLA risk group 3	185 (19.0)	65.4	483 (25.3)	62.9
HLA risk group 4	501 (51.3)	59.9	932 (48.8)	59.1
HLA risk group 5	245 (25.1)	56.3	229 (12.0)	55.9
Other^a^	6 (0.6)	33.3	72 (3.8)	50.0
Sum	976	59.5	1910	59.7

B	IAA first (%)	Males (%)	GADA first (%)	Males (%)
Age at seroconversion, years				
≤2	174 (52.7)	57.5	67 (20.4)	68.7
>2 and ≤4	76 (23.0)	52.6	79 (24.0)	59.5
>4 and ≤6	27 (8.2)	59.3	69 (21.0)	53.6
>6 and ≤10	36 (10.9)	63.9	78 (23.7)	61.5
>10 and ≤15	17 (5.2)	52.9	36 (10.9)	61.1
Sum	330	57.0	329	60.8

^a^ HLA-genotypes which have no defined HLA, risk group.

### 2.2 Genotyping

The eligibility criteria of the DIPP study have been modified since its launch in 1994 when only a limited set of HLA-DQB1 alleles was analyzed (Ilonen et al., 2016). As of now, HLA typing is performed with an in-house DELFIA-based system for HLA-DRB1 typing (Kiviniemi et al., 2007), and an in-house homogenous PCR assay for HLA-DQB1 and HLA-DQA1 typing (Kiviniemi et al., 2007). Children eligible for the study are either homozygous for the (DR3)-DQA1*05-DQB1*02 (DR3-DQ2) haplotype or the DRB1*04:01/2/4/5-DQA1*03-DQB1*03:02 (DR4-DQ8) haplotype, or alternatively carry the combined DR3-DQ2/DR4-DQ8 genotype. Moreover, those who carry the DR3-DQ2/(DR9)-DQA1*03-DQB1*03:03 (DR9-DQ9) genotype or the high-risk DR4-DQ8 haplotype in combination with the (DR1/10)-DQB1*05:01 (DR1-DQ5), (DR7)-DQA1*02:01-DQB1*02 (DR7-DQ2), (DR8)-DQB1*04 (DR8-DQ4), DR9-DQ9, or (DR13)-DQB1*06:04 (DR13-DQ6.4) haplotypes are also eligible.

Regarding the analyzed HLA risk groups, all considered haplotypes either confer strong susceptibility (S), intermediate susceptibility (s), strong protection (P), weak protection (p), or exert a neutral effect (N) (Ilonen et al., 2016). Based on this definition, strong susceptibility (S) is defined by DRB1*04:01/2/5-DQA1*03-DQB1*03:02, whereas DRB1*04:04-DQA1*03-DQB1*03:02 and (DR3)-DQA1*05-DQB1*02 confer weaker susceptibility (s). The haplotypes defined as neutral (N) are (DR1/10)-DQB1*05:01, (DR13)-DQB1*06:04/9, (DR16)-DQB1*05:02, (DR4)-DQA1*03-DQB1*03:01, (DR7)-DQA1*02:01-DQB1*02, (DR9)-DQA1*03-DQB1*03:03, and (DR8)-DQB1*04. Strong protection (P) is conferred by (DR7)-DQA1*02:01-DQB1*03:03, (DR15)- DQB1*06:01/2 and (DR14)-DQB1*05:03, while weak protection (p) is conferred by DRB1*04:03-DQA1*03-DQB1*03:02, (DR13)-DQB1*06:03, and (DR11/12/13)-DQA1*05-DQB1*03:01.

The high risk group (5) is formed by those who carry any combination of DR3-DQ2 with DR4-DQ8 (S/s and s/s). The intermediate risk group (4) is formed by all other S/s than the DR3-DQ2/DR4-DQ8 combination, with the addition of the remaining S/S, s/s, and S/N genotypes. The group with slightly increased risk (3) is defined by the S/p and s/N genotypes, whereas the neutral risk group (2) is defined by the N/N, S/P, s/P, and s/p combinations. Lastly, the group with slightly decreased risk (1) is defined by p/N genotypes, and the remaining P/N, p/p, P/p, and P/P genotypes form the group with greatly decreased risk (0).

For this study, 2886 DIPP subjects were genotyped for the *CXADR* SNPs with the QuantStudio 3 Real-Time PCR platform (ThermoFisher; cat. A28317) using a commercial TaqMan probe for rs2824404 (ThermoFisher, assay ID; C___3051168_10) and a custom-made probe for rs6517774 (ThermoFisher). The rs6517774 probes were designed with ThermoFisher’s public TaqMan Assay Design Tool according to the following specifications: a 5′-[VIC]CAAAGTATCAGCCCCTAGT[NFQ]-3′ A-allele probe, and a 5′-[FAM]AAGTATCGGCCCCTAGTT[NFQ]-3′ G-allele probe. The strategy for sample preparation, TaqMan genotyping and results analysis has been published earlier (Nygård et al., 2021).

### 2.3 Measurement of type 1 diabetes-associated autoantibodies

Serotyping for GADA, IA-2A, IAA, and ZnT8A was conducted with serum or plasma in an AAb-specific radio-binding assay after each scheduled follow-up visit (Siljander et al., 2009; Juusola et al., 2016). The standardized limits for seropositivity have been described earlier (Juusola et al., 2016; Lamichhane et al., 2023).

### 2.4 Statistical analysis

A descriptive analysis was conducted to determine the frequency distribution of the genotypes. The deviation in Hardy-Weinberg equilibrium was calculated using Chi-square tests, and a *p* value < 0.05 was considered significant. To determine the impact of the genotyped SNP on the age at diagnosis and age at seroconversion, a survival analysis (Cox regression proportional hazard model) was performed with IBM SPSS 27 software. The analyzed time intervals were from birth to seroconversion, from seroconversion to T1D diagnosis, and from birth to T1D diagnosis. The analysis was conducted according to recessive, additive and dominant inheritance models with rs6517774-A and rs2824404-T set as reference alleles. The models were applied separately in cases with IAA as the first AAb, in cases with GADA as the first AAb and in all groups combined. Moreover, respective models were adjusted with HLA risk groups and sex. All models were procured in a forward fashion, starting from the SNP, then adding the HLA risk groups, and lastly the sex. The statistical approach applied in this paper has been described earlier (Laine et al., 2022). Figures were made with GraphPad Prism 9.2 software.

## 3 Results

There were no significant differences found in the frequency distribution of rs2824404 and rs6517774 in case-control pairs, with cases having either one or more AAbs (Supplementary Table S1) or a T1D diagnosis (Supplementary Table S2). Subsequently, a stratified analysis of all groups (seropositive non-diabetic cases and seropositive T1D cases, respectively, against healthy controls) was conducted for respective sex (Supplementary Tables S3–S6). When analyzing the distribution of the rs6517774 variant in males and females separately, significant yet less concordant differences were observed (Supplementary Tables S3–S6). The distribution of the rs6517774 SNP was further analyzed with the intention to detect differences in allelic distribution between sexes in seropositive cases and in healthy subjects (Table 2). When comparing allele frequencies between seropositive males and females, a significant divergence was discovered in seropositive cases, as the rs6517774-A allele was significantly more common in seropositive females than in seropositive males (Table 2).

**TABLE 2:** rs6517774 (A/G) exhibits sex-disparity in seropositive genotypes. The upper block includes healthy seronegative subject and depicts the allelic distribution of the rs6517774 SNPs in males and females. Similarly, the lower block includes autoantibody positive subjects (AAb+) with the addition of a dominant inheritance model where the alternate allele is rs6517774-G. Significant *p* values (*p* < 0.05) are bolded.

			Female	Male	χ2 test		Total
			N (%)	N (%)	OR (95% CI)	*p* value	N (%)
Healthy subjects	rs6517774	AA	267 (34.8)	408 (36.3)	0.94 (0.77–1.13)	0.498	675 (35.7)
	(A/G)	AG	394 (51.4)	554 (49.3)	1.08 (0.90–1.30)	0.384	948 (50.2)
		GG	106 (13.8)	161 (14.3)	0.96 (0.74–1.25)	0.752	267 (14.1)
	Total		767	1123			1890
	Alleles	A	928 (60.5)	1370 (61.0)	0.98 (0.86–1.12)	0.756	2298 (60.8)
		G	606 (39.5)	876 (39.0)	1.02 (0.89–1.25)	0.756	1482 (39.2)
AAb + subjects	rs6517774	AA	162 (41.4)	177 (31.3)	1.55 (1.19–2.03)	**0.001**	339 (35.4)
	(A/G)	AG	170 (43.5)	296 (52.3)	0.70 (0.54–0.91)	**0.007**	466 (48.7)
		GG	59 (15.1)	93 (16.4)	0.90 (0.63–1.29)	0.577	152 (15.9)
	Total		391	566			957
	Alleles	A	494 (63.2)	650 (57.4)	1.27 (1.05–1.53)	**0.012**	1144 (59.8)
		G	288 (36.8)	482 (42.6)	0.79 (0.65–0.95)	**0.012**	770 (40.2)
	Dominant model	AA	162 (41.4)	117 (31.3)	1.55 (1.19–2.03)	**0.001**	339 (35.4)
		AG + GG	229 (58.6)	389 (68.7)	0.64 (0.49–0.84)	**0.001**	618 (64.6)

**FIGURE 1:**
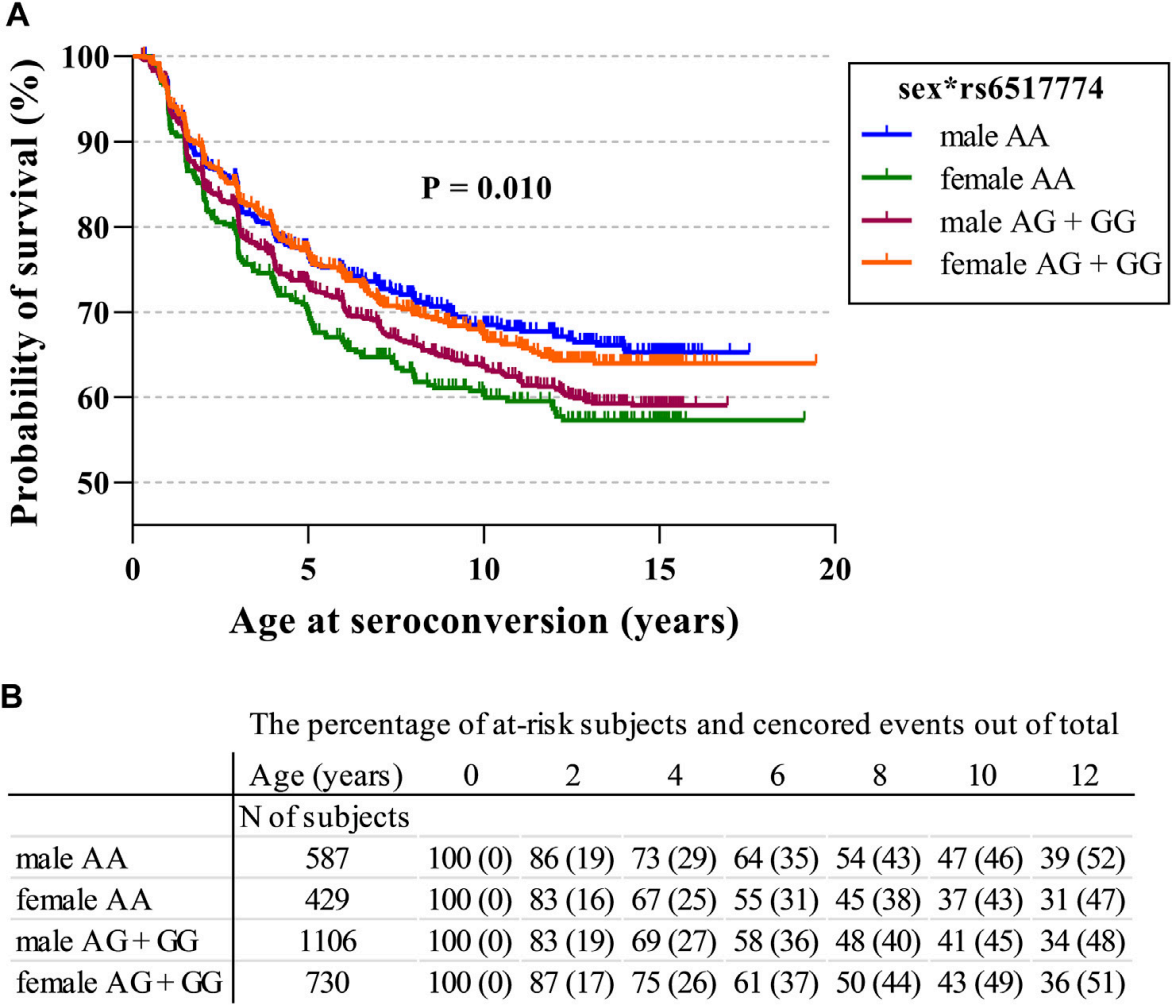
*CXADR* rs6517774 (A/G) is associated with islet autoimmunity in a sex-dependent manner. **(A)**. The Kaplan-Meier figure depicts the probability of survival to seroconversion for respective sex in conjunction with the rs6517774 genotype (dominant inheritance model: AA vs. AG + GG). Log-rank test reports a significant difference between groups (*p* = 0.010, χ^2^ = 11.40, df = 3) **(B)**. The table lists the remaining fraction of at-risk subjects (out of the total subject number) and the fraction of censored events (out of the total event number) at each time point. Right-censored events mark the last recorded datapoint of seronegative subjects who have excited the follow-up.

A Cox regression survival analysis revealed that homozygosity for the rs6517774-A allele (AA vs. AG + GG; HR = 0.81, *p* = 0.022, 95% CI = 0.68–0.97) and the female sex (HR = 0.84, *p* = 0.041, 95% CI = 0.72–0.99) were associated with a later age at seroconversion (Table 3). Moreover, the rs6517774 genotype exhibited in a dominant manner (Female*AA vs. Other) an interaction with the sex that yielded a younger age at seropositivity in females (HR = 1.53, *p* = 0.002, 95% CI = 1.17–2.01) concurrent to an increased time to seropositivity in males (Figure 1; observe the directions of effects). Lastly, in the Cox regression survival analysis, no corroborative differences were found when analyzing rs2824404 in a similar manner to rs6517774.

**TABLE 3:** Summary of the results of the Cox regression analyses. Table presents data from the most fit model where the effects of the rs6517774 SNP on respective time-to-event is adjusted by HLA risk groups and sex. The subgroup analysis of IAA-first and GADA-first serotypes did not yield significant results. All models were tested according to a recessive, additive and dominant inheritance model, although only the dominant model (AA vs. AG + GG) yielded notable results. Significant *p* values (*p* < 0.05) are bolded.

Variable	Reference	Comparison	Birth to first AAB			First AAB to T1D			Birth to T1D		
			*p* value	HR	95% CI	*p* value	HR	95% CI	*p* value	HR	95% CI
rs6517774	AG + GG	AA	**0.022**	0.81	0.68–0.97	0.378	1.13	0.86–1.48	0.410	0.89	0.68–1.17
sex	Male	Female	**0.041**	0.84	0.72–0.99	0.124	1.22	0.95–1.56	0.909	0.99	0.77–1.26
rs6517774*sex	Other	Female*AA	**0.002**	1.53	1.17–2.01	0.564	1.12	0.76–1.67	**0.033**	1.54	1.04–2.92
Risk group 3	Other	Group 3	**<0.001**	1.88	1.33–2.65	**0.036**	2.30	1.06–5.03	**0.001**	3.81	1.75–8.30
Risk group 4	Other	Group 4	**<0.001**	2.74	1.98–3.80	**0.006**	2.90	1.36–6.16	**<0.001**	6.70	3.15–14.23
Risk group 5	Other	Group 5	**<0.001**	4.61	3.29–6.48	**<0.001**	4.79	2.24–10.25	**<0.001**	14.73	6.89–31.48

## 4 Discussion

The TEDDY study reported that the minor rs6517774-G allele was significantly more common in cases with IA than in their matched controls (Vehik et al., 2019). The investigators reported a SNP case-control frequency difference of six percentage points. However, we could only see a one-percentage-point difference in DIPP participants (Supplementary Table S1). Vehik et al. also reported, that the minor rs2824404-C allele was more frequent in Finnish children with IA when adjusted for HLA-genotype. The previous study observed a rs2824404-C case-control difference of 3.3-percentage-points. In the present study, the difference was 2.7-percentage-points with the C-allele being more frequent in the controls (Supplementary Table S1).

According to the SNP database of the NCBI (Sayers et al., 2022), rs6517774 is a non-coding variant located 63 kb upstream of the *CXADR* gene. Rs2824404, on the other hand, is either classified as a non-coding downstream variant or a 3′-UTR variant due to the *CXADR* gene encoding multiple transcripts. By enquiring RegulomeDB (Dong et al., 2023) and FORGEdb (Breeze et al., 2023), one can assess that the functional annotations for both SNPs are limited to differential-expression data unrelated to immunological pathways. With this in mind, we would reason that rs2824404 do not contribute with an independent risk effect in Finnish individuals. Especially since there is no linkage (*D*′ = 0.025, *R* ^2^ = 0.0) between the two SNPs, as reported by LDlink using the Finnish 1000Genomes excerpt (Machiela and Chanock, 2015), and since all statistical tests for rs2824404 were made in parallel with the tests for rs6517774.

Reverting to the main results, novel and significant rs6517774-mediated differences were found by stratifying the analysis of seropositive cases according to sex. Nonetheless, the results were difficult to interpret since the effects changed direction in conjunction with the sex (Supplementary Tables S3–S6). Subsequently, a sex disparity was discovered by comparing the distribution of rs6517774 alleles in seropositive females against seropositive males; a difference absent in healthy subjects (Table 2). The analysis revealed a 10-percentage-point higher frequency of the rs6517774-AA genotype in seropositive females, when compared to seropositive males; a result that would point towards the presence of a SNP-sex interaction in seropositive subjects.

To further corroborate these results, a multivariate survival analysis was conducted with the intention of searching for a *CXADR* SNP-mediated effect on the age at seroconversion or age at T1D diagnosis (Table 3). The procured model was fitted with sex and HLA risk groups as covariates so as to account for the effects of the HLA background and to confirm the presence of a sex disparity. By accounting for the impact that HLA risk groups has on survival times, novel SNP-mediated effects were identified. First, homozygosity for the rs6517774-A allele (according to a dominant AA vs. AG + GG model) exhibited an independent effect (HR = 0.81, 95% CI = 0.68–0.97) that increased the age at seroconversion. Secondly, females had a significantly later age at seroconversion (HR = 0.84, 95% CI = 0.72–0.99) as compared to males. And thirdly, rs6517774 displayed a significant interaction with sex (annotated rs6517774*sex), as the females carrying the rs6517774-AA genotype had a shorter survival time to seroconversion (HR = 1.53, 95% CI = 1.17–2.01) and to T1D (HR = 1.54, 95% CI = 1.04–2.92). Notably, the interaction entails that the opposite effect occurs in males, thus implying an increased survival to the aforementioned events (Figure 1).

Consequently, the association of the rs6517774 SNP with IA can be explained by the existence of a SNP-sex interaction that modifies the time-to-event survival for IA. However, the effects only emerge when accounting for the HLA background and the sex. Moreover, since the rs6517774, sex and rs6517774*sex variables lack a significant association with progression time from seropositivity to T1D diagnosis (Table 3), we suggest that the impact of the SNP is limited to IA induction.

The survival analysis (Table 3) also shows that females have a later age at seroconversion; an observation supported by past observations made in DIPP and TEDDY cohorts. For example, the multinational TEDDY study recently reported that the male sex is associated with IA, whereas sex did not affect the age at seroconversion nor T1D diagnosis, except by increasing the progression rate from AAb multipositivity to T1D (Krischer et al., 2022). However, in a previous report applying a different multivariate model, the female sex was associated with progression from multipositivity to T1D, while the male sex alone affected the age at IA initiation (Krischer et al., 2017). Moreover, the sex seemingly affects time-to-event characteristics in a serotype-specific manner. The DIPP study recently reported that GADA-initiated IA was more common in males, while IAA-initiated IA was more common in females (Ilonen et al., 2022). Although, the connection between sex, serological subgroups, and risk for IA and T1D is evidently complex. In our dataset, the sex does not by itself significantly affect the age at seroconversion; however, the sex becomes a good variable when accounting for the effects mediated by HLA risk groups. It is the addition of rs6517774, and then rs6517774*sex, which potentiates the sex as a variable. Nonetheless, a separate Kaplan-Meier survival analysis of various serotype groups did detect differences based on sex and the rs6517774 SNP (Supplementary Table S7), but these differences cannot be reliably separated from the HLA genotypes’ independent associations to specific serotype groups. Here, the most prominent effect is still that rs6517774-AA (AA vs. AG + GG), in a sex-dependent manner, modify the age at seroconversion and T1D in subjects with one or more AAbs. This study’s observations cannot be further corroborated without accounting for other factors heavily intertwined with the sex, such as the familial history of T1D, the presence of high-risk SNPs (e.g., INS and PTPN22), and especially the viral background which we postulate to be the main environmental risk factor interacting with the CXADR.

Recently, Li et al. conducted a GWAS analysis of an European lung cancer cohort and found that a low-frequency *CXADR* SNP (rs208908) affected the risk for lung cancer in a sex-dependent manner (Li et al., 2022). Moreover, the authors suggested that the cause of this sex disparity stemmed from sex-based differential expression. However, in our research, it can only be speculated whether our findings affect one or more of the biological functions of CXADR due to the protein’s multifaceted nature as a structural receptor and virus receptor. The data repository of the DIPP study is limited to genetic and serological data, and, thus, we cannot account for the viral environmental background which might explain how rs6517774 generates a sex-dependent effect on IA induction. We would also encourage that *CXADR* gene expression is explored on a tissue-specific level in conjunction with sex and the rs6517774 SNP.

To conclude, the rs6517774-G SNP was previously reported to produce an additive risk effect for IA concurrent to a decrease in enteroviral infection rates. In this study, with HLA-risk groups and sex set as covariates, a Cox regression survival analysis revealed that homozygosity for rs6517774-A was associated with a younger age at seroconversion in females (female*AA), while introducing a protective effect in males. Our results, visually outlined in Figure 1, indicate that the rs6517774 SNP alters IA characteristics by modifying the age at seroconversion in a sex-dependent manner.

## Data Availability

The datasets presented in this article are not readily available due to data privacy regulations. Requests to access the datasets should be directed via the corresponding author to the DIPP Steering Committee.
