# Supplementary material for: CXADR polymorphism rs6517774 modifies islet autoimmunity characteristics and exhibits sex disparity
Source: Front Genet. 2023 Nov 2;14:1248701. doi: 10.3389/fgene.2023.1248701 (PMC10651746; doi:10.3389/fgene.2023.1248701)
Supplement: Supplementary file 1 [file Table1.docx]

Supplementary Material

CXADR polymorphism rs6517774 modifies islet autoimmunity characteristics and exhibits sex disparity

Lucas Nygård^*^, Milla Valta, Antti-Pekka Laine, Jorma Toppari, Mikael Knip, Riitta Veijola, Heikki Hyöty, Jorma Ilonen, Johanna Lempainen

*** Correspondence:** Lucas Nygård, nygard@uef.fi

# Supplementary Tables

Supplementary Table 1. Distribution of rs2824404 (T/C) and rs6517774 (A/G) alleles in AAb-positive pairs. Cases were positive for one or more AAbs.

|  |  | **Cases** | **Controls** | **χ2 test** |  | **Total** |
| --- | --- | --- | --- | --- | --- | --- |
|  |  | **N (%)** | **N (%)** | **OR (95% CI)** | **P value** | **N (%)** |
| **rs2824404** | TT | 261 (27.1) | 464 (24.5) | 1.15 (0.96–1.37) | 0.132 | 725 (25.3) |
| **(T/C)** | CT | 495 (51.4) | 970 (51.2) | 1.01 (0.86–1.18) | 0.935 | 1465 (51.3) |
|  | CC | 207 (21.5) | 459 (24.2) | 0.86 (0.71–1.03) | 0.100 | 666 (23.3) |
| **Total** |  | 963 | 1893 |  |  | 2856 |
| **Alleles** | T | 1017 (52.8) | 1898 (50.1) | 1.11 (1.00–1.24) | 0.056 | 2915 (51.0) |
|  | C | 909 (47.2) | 1888 (49.9) | 0.90 (0.81–1.00) | 0.056 | 2797 (49.0) |
| **rs6517774** | AA | 339 (35.4) | 675 (35.7) | 0.99 (0.84–1.16) | 0.878 | 1014 (35.6) |
| **(A/G)** | AG | 466 (48.7) | 948 (50.2) | 0.94 (0.81–1.10) | 0.460 | 1414 (49.7) |
|  | GG | 152 (15.9) | 267 (14.1) | 1.15 (0.92–1.42) | 0.212 | 419 (14.7) |
| **Total** |  | 957 | 1890 |  |  | 2847 |
| **Alleles** | A | 1144 (59.8) | 2298 (60.8) | 0.96 (0.86–1.07) | 0.456 | 3442 (60.4) |
|  | G | 770 (40.2) | 1482 (39.2) | 1.04 (0.93–1.17) | 0.456 | 2252 (39.6) |

Supplementary Table 2. Distribution of rs2824404 (T/C) and rs6517774 (A/G) alleles in subjects with T1D diagnosis. Cases were diagnosed with T1D and the controls were healthy and AAb negative.

|  |  | **Cases** | **Controls** | **χ2 test** |  | **Total** |
| --- | --- | --- | --- | --- | --- | --- |
|  |  | **N (%)** | **N (%)** | **OR (95% CI)** | **P value** | **N (%)** |
| **rs2824404** | TT | 119 (28.3) | 464 (24.5) | 1.22 (0.96–1.54) | 0.103 | 583 (25.2) |
| **(T/C)** | CT | 206 (49.0) | 970 (51.2) | 0.92 (0.74–1.13) | 0.416 | 1176 (50.8) |
|  | CC | 95 (22.6) | 459 (24.2) | 0.91 (0.71–1.17) | 0.479 | 554 (24.0) |
| **Total** |  | 420 | 1893 |  |  | 2313 |
| **Alleles** | T | 444 (52.9) | 1898 (50.1) | 1.12 (0.96–1.30) | 0.153 | 2342 (50.6) |
|  | C | 396 (47.1) | 1888 (49.9) | 0.90 (0.77–1.04) | 0.153 | 2284 (49.4) |
| **rs6517774** | AA | 160 (38.2) | 675 (35.7) | 1.11 (0.89–1.38) | 0.341 | 835 (36.2) |
| **(A/G)** | AG | 206 (49.2) | 948 (50.2) | 0.96 (0.78–1.19) | 0.713 | 1154 (50.0) |
|  | GG | 53 (12.6) | 267 (14.1) | 0.88 (0.64–1.21) | 0.428 | 320 (13.9) |
| **Total** |  | 419 | 1890 |  |  | 2309 |
| **Alleles** | A | 526 (62.8) | 2298 (60.8) | 1.09 (0.93–1.27) | 0.289 | 2824 (61.2) |
|  | G | 312 (37.2) | 1482 (39.2) | 0.92 (0.79–1.07) | 0.289 | 1794 (38.8) |

Supplementary Table 3. Distribution of rs2824404 (T/C) and rs6517774 (A/G) alleles in AAb-positive females. Cases were positive for one or more AAbs.

|  |  | **Cases** | **Controls** | **χ2 test** |  | **Total** |
| --- | --- | --- | --- | --- | --- | --- |
|  |  | **N (%)** | **N (%)** | **OR (95% CI)** | **P value** | **N (%)** |
| **rs2824404** | TT | 117 (29.8) | 182 (23.7) | 1.37 (1.04–1.80) | **0.024** | 299 (25.8) |
| **(T/C)** | CT | 198 (50.5) | 398 (51.8) | 0.95 (0.74–1.21) | 0.672 | 596 (51.4) |
|  | CC | 77 (19.6) | 188 (24.5) | 0.75 (0.56–1.02) | 0.063 | 265 (22.8) |
| **Total** |  | 392 | 768 |  |  | 1160 |
| **Alleles** | T | 432 (55.1) | 762 (49.6) | 1.25 (1.05–1.48) | **0.012** | 1194 (51.5) |
|  | C | 352 (44.9) | 774 (50.4) | 0.80 (0.67–0.95) | **0.012** | 1126 (48.5) |
| **Dominant model** | TT | 117 (29.8) | 182 (23.7) | 1.37 (1.04–1.80) | **0.024** | 299 (25.8) |
|  | CC+CT | 275 (70.2) | 586 (76.3) | 0.73 (0.56–0.96) | **0.024** | 861 (74.2) |
| **rs6517774** | AA | 162 (41.4) | 267 (34.8) | 1.32 (0.76–1.23) | 0.765 | 429 (37.0) |
| **(A/G)** | AG | 170 (43.5) | 394 (51.4) | 0.73 (0.57–0.93) | **0.011** | 564 (48.7) |
|  | GG | 59 (15.1) | 106 (13.8) | 1.11 (0.79–1.56) | 0.559 | 165 (14.2) |
| **Total** |  | 391 | 767 |  |  | 1158 |
| **Alleles** | A | 494 (63.2) | 928 (60.5) | 1.12 (0.94–1.34) | 0.211 | 1422 (61.4) |
|  | G | 288 (36.8) | 606 (39.5) | 0.89 (0.75–1.07) | 0.211 | 894 (38.6) |
| **Dominant model** | AA | 162 (41.4) | 267 (34.8) | 1.32 (1.03–1.70) | **0.027** | 429 (37.0) |
|  | AG+GG | 229 (58.6) | 500 (65.2) | 0.75 (0.59–0.97) | **0.027** | 729 (63.0) |

Supplementary Table 4. Distribution of rs2824404 (T/C) and rs6517774 (A/G) alleles in AAb-positive males. Cases were positive for one or more AAbs.

|  |  | **Cases** | **Controls** | **χ2 test** |  | **Total** |
| --- | --- | --- | --- | --- | --- | --- |
|  |  | **N (%)** | **N (%)** | **OR (95% CI)** | **P value** | **N (%)** |
| **rs2824404** | TT | 144 (25.2) | 282 (25.1) | 1.01 (0.80–1.27) | 0.946 | 426 (25.1) |
| **(T/C)** | CT | 297 (52.0) | 572 (50.8) | 1.05 (0.86–1.28) | 0.649 | 869 (51.2) |
|  | CC | 130 (22.8) | 271 (24.1) | 0.93 (0.73–1.18) | 0.545 | 401 (23.6) |
| **Total** |  | 571 | 1125 |  |  | 1696 |
| **Alleles** | T | 585 (51.2) | 1136 (50.5) | 1.03 (0.89–1.19) | 0.685 | 1721 (50.7) |
|  | C | 557 (48.8) | 1114 (49.5) | 0.97 (0.84–1.12) | 0.685 | 1671 (49.3) |
| **Dominant model** | TT | 144 (25.2) | 282 (25.1) | 1.01 (0.80–1.27) | 0.946 | 426 (25.1) |
|  | CC+CT | 427 (74.8) | 843 (74.9) | 0.99 (0.79–1.25) | 0.946 | 1270 (74.9) |
| **rs6517774** | AA | 177 (31.3) | 408 (36.3) | 0.80 (0.64–0.99) | **0.039** | 585 (34.6) |
| **(A/G)** | AG | 296 (52.3) | 554 (49.3) | 1.13 (0.92–1.38) | 0.250 | 850 (50.3) |
|  | GG | 93 (16.4) | 161 (14.3) | 1.17 (0.89–1.55) | 0.256 | 254 (15.0) |
| **Total** |  | 566 | 1123 |  |  | 1689 |
| **Alleles** | A | 650 (57.4) | 1370 (61.0) | 0.86 (0.75–1.00) | **0.045** | 2020 (59.8) |
|  | G | 482 (42.6) | 876 (39.0) | 1.16 (1.00–1.34) | **0.045** | 1358 (40.2) |
| **Dominant model** | AA | 177 (31.3) | 408 (36.3) | 0.80 (0.64–0.99) | **0.039** | 585 (34.6) |
|  | AG+GG | 389 (68.7) | 715 (63.7) | 1.25 (1.01–1.56) | **0.039** | 1104 (65.4) |

Supplementary Table 5. Distribution of rs2824404 (T/C) and rs6517774 (A/G) alleles in female subjects with T1D. Cases were diagnosed with T1D and the controls were healthy and AAb negative.

|  |  | **Cases** | **Controls** | **χ2 test** |  | **Total** |
| --- | --- | --- | --- | --- | --- | --- |
|  |  | **N (%)** | **N (%)** | **OR (95% CI)** | **P value** | **N (%)** |
| **rs2824404** | TT | 60 (32.4) | 182 (23.7) | 1.55 (1.09–2.19) | **0.014** | 242 (25.4) |
| **(T/C)** | CT | 83 (44.9) | 398 (51.8) | 0.76 (0.55–1.04) | 0.089 | 481 (50.5) |
|  | CC | 42 (22.7) | 188 (24.5) | 0.91 (0.62–1.33) | 0.612 | 230 (24.1) |
| **Total** |  | 185 | 768 |  |  | 953 |
| **Alleles** | T | 203 (54.9) | 762 (49.6) | 1.23 (0.98–1.55) | 0.070 | 965 (50.6) |
|  | C | 167 (45.1) | 774 (50.4) | 0.81 (0.64–1.02) | 0.070 | 941 (49.4) |
| **Dominant model** | TT | 60 (32.4) | 182 (23.7) | 1.55 (1.09–2.19) | **0.014** | 242 (25.4) |
|  | CC+CT | 125 (67.6) | 586 (76.3) | 0.65 (0.46–0.92) | **0.014** | 711 (74.6) |
| **rs6517774** | AA | 81 (44.0) | 267 (34.8) | 1.47 (1.06–2.04) | **0.020** | 348 (36.6) |
| **(A/G)** | AG | 79 (42.9) | 394 (51.4) | 0.71 (0.51–0.99) | **0.040** | 473 (49.7) |
|  | GG | 24 (13.0) | 106 (13.8) | 0.94 (0.58–1.50) | 0.783 | 130 (13.7) |
| **Total** |  | 184 | 767 |  |  | 951 |
| **Alleles** | A | 241 (65.5) | 928 (60.5) | 1.24 (0.98–1.57) | 0.077 | 1169 (61.5) |
|  | G | 127 (34.5) | 606 (39.5) | 0.81 (0.64–1.00) | 0.077 | 733 (38.5) |
| **Dominant model** | AA | 81 (44.0) | 267 (34.8) | 1.47 (1.06–2.04) | **0.020** | 348 (36.6) |
|  | AG+GG | 103 (56.0) | 500 (65.2) | 0.68 (0.49–0.94) | **0.020** | 603 (63.4) |

Supplementary Table 6. Distribution of rs2824404 (T/C) and rs6517774 (A/G) alleles in male subjects with T1D. Cases were diagnosed with T1D and the controls were healthy and AAb negative.

|  |  | **Cases** | **Controls** | **χ2 test** |  | **Total** |
| --- | --- | --- | --- | --- | --- | --- |
|  |  | **N (%)** | **N (%)** | **OR (95% CI)** | **P value** | **N (%)** |
| **rs2824404** | TT | 59 (25.1) | 282 (25.1) | 1.00 (0.72–1.39) | 0.990 | 341 (25.1) |
| **(T/C)** | CT | 123 (52.3) | 572 (50.8) | 1.06 (0.80–1.41) | 0.676 | 695 (51.1) |
|  | CC | 53 (22.6) | 271 (24.1) | 0.92 (0.66–1.28) | 0.615 | 324 (23.8) |
| **Total** |  | 235 | 1125 |  |  | 1360 |
| **Alleles** | T | 241 (51.3) | 1136 (50.5) | 1.03 (0.85–1.26) | 0.756 | 1377 (50.6) |
|  | C | 229 (48.7) | 1114 (49.5) | 0.97 (0.79–1.18) | 0.756 | 1343 (49.4) |
| **Dominant model** | TT | 59 (25.1) | 282 (25.1) | 1.00 (0.72–1.39) | 0.990 | 341 (25.1) |
|  | CC+CT | 176 (74.9) | 843 (74.9) | 1.00 (0.72–1.38) | 0.990 | 1019 (74.9) |
| **rs6517774** | AA | 79 (33.6) | 408 (36.3) | 0.89 (0.66–1.19) | 0.430 | 487 (35.9) |
| **(A/G)** | AG | 127 (54.0) | 554 (49.3) | 1.21 (0.91–1.60) | 0.189 | 681 (50.1) |
|  | GG | 29 (12.3) | 161 (14.3) | 0.84 (0.55–1.22) | 0.422 | 190 (14.0) |
| **Total** |  | 235 | 1123 |  |  | 1358 |
| **Alleles** | A | 285 (60.6) | 1370 (61.0) | 0.99 (0.80–1.21) | 0.885 | 1655 (60.9) |
|  | G | 185 (39.4) | 876 (39.0) | 1.02 (0.83–1.24) | 0.885 | 1061 (39.1) |
| **Dominant model** | AA | 79 (33.6) | 408 (36.3) | 0.89 (0.66–1.19) | 0.430 | 487 (35.9) |
|  | AG+AG | 156 (66.4) | 715 (63.7) | 1.13 (0.84–1.52) | 0.430 | 871 (64.1) |

Supplementary Table 7. *CXADR* rs6517774 (A/G) modifies progression rate to islet-autoimmunity and T1D in a sex-dependent manner. Summary table of Kaplan-Meier Log-rank test scores. The survival analysis tested for rs6517774-mediated effects (dominant model: AA vs. AG+GG) on progression rate from birth to seroconversion, seroconversion to T1D diagnosis, and from birth to T1D diagnosis. The analysis was conducted separately in subjects with one AAb, more than one AAb, and one or more detected AAbs during follow-up. Similarly, the analysis was made separately in subjects with a single AAb at seroconversion, multiple AAbs at seroconversion, and then in subjects with a specific AAb that appeared alone. All time-to-event–group comparisons where stratified according to sex, and the p-value is reported for each stratum. All significant groupwise differences in survival show that rs6517774-AA (AA vs. AG+GG) lower the time-to-event survival in females, while increasing the time to event survival in males. A P value <0.05 was considered significant.

|  | **During follow-up** | | | **At seroconversion** | | | | |
| --- | --- | --- | --- | --- | --- | --- | --- | --- |
| **rs6517774**  **(AA vs AG+GG)** | **One or more AAbs** | **One AAb** | **More than one AAb** | **Single AAb** | **Multiple AAbs** | **IAA alone** | **GADA alone** | **IA2A alone** |
| **Birth to seroconversion** |  |  |  |  |  |  |  |  |
| Male | **0.024** | 0.220 | 0.055 | 0.062 | 0.199 | 0.508 | 0.264 | 0.342 |
| Female | **0.012** | 0.054 | 0.097 | **0.029** | 0.212 | **0.039** | 0.403 | **0.036** |
| **Seroconversion to T1D** |  |  |  |  |  |  |  |  |
| Male | 0.096 | 0.154 | 0.335 | 0.093 | 0.683 | 0.104 | 0.192 | 0.249 |
| Female | 0.141 | 0.202 | 0.398 | 0.164 | 0.592 | 0.092 | 0.879 | 0.511 |
| **Birth to T1D** |  |  |  |  |  |  |  |  |
| Male | **0.039** | 0.261 | 0.078 | 0.085 | 0.248 | 0.516 | 0.367 | **0.035** |
| Female | **0.007** | **0.038** | 0.078 | **0.021** | 0.172 | **0.026** | 0.402 | 0.321 |
